# The Biosimilar Landscape: An Overview of Regulatory Approvals by the EMA and FDA

**DOI:** 10.3390/pharmaceutics13010048

**Published:** 2020-12-31

**Authors:** Ioana Gherghescu, M. Begoña Delgado-Charro

**Affiliations:** Department of Pharmacy and Pharmacology, University of Bath, Bath BA2 7AY, UK; ioana.gherghescu@bath.edu

**Keywords:** biosimilars, FDA, EMA, adalimumab, Humira, biosimilarity

## Abstract

Biosimilar medicines expand the biotherapeutic market and improve patient access. This work looked into the landscape of the European and US biosimilar products, their regulatory authorization, market availability, and clinical evaluation undergone prior to the regulatory approval. European Medicines Agency (EMEA, currently EMA) and Food and Drug Administration (FDA) repositories were searched to identify all biosimilar medicines approved before December 2019. Adalimumab biosimilars, and particularly their clinical evaluations, were used as a case study. In the past 13 years, the EMA has received 65 marketing authorization applications for biosimilar medicines with 55 approved biosimilars available in the EU market. Since the first biosimilar approval in 2015, the FDA has granted 26 approvals for biosimilars with only 11 being currently on the US market. Five adalimumab biosimilars have been approved in the EU and commercialized as eight different medicines through duplicate marketing authorizations. Whilst three of these are FDA-approved, the first adalimumab biosimilar will not be marketed in the US until 2023 due to Humira’s exclusivity period. The EU biosimilar market has developed faster than its US counterpart, as the latter is probably challenged by a series of patents and exclusivity periods protecting the bio-originator medicines, an issue addressed by the US’s latest ‘Biosimilar Action Plan’.

## 1. Introduction

Biological therapies have revolutionized care for many patients with chronic conditions, whilst putting a financial strain on healthcare systems that provide these expensive therapies and, in some instances, on patients themselves. As patents and exclusivity periods for the bio-originators expire, the development of biosimilar medicines can grow the biotherapeutic market and improve patient outcomes by facilitating access to biological therapies. According to Mehr, “these emerging biotherapeutics are perceived as major tools to control cost and increase access to biologic drugs” [1]. A 2019 IQVIA (Durham, NC, USA) report produced at the request of the European Commission has summarized the impact of biosimilar competition in European markets [2].

Biosimilars (BS) are biological medicines that are highly similar to an already-approved biologic, called the reference product (RP). The World Health Organization (WHO) refers to them as similar biotherapeutic products (SBPs) and defines them as ‘highly similar to an original biotherapeutic product’ [3] and ‘developed and assessed according to the regulatory guidelines that ensure an adequate comparison of the SBP with its RBP’ [3].

The regulatory approval pathway for biosimilar medicines at EU level was first developed in 2005, enabling the European Medicines Agency (EMEA, currently EMA) to approve the first biosimilar, Omnitrope, in 2006 [4]. The US pathway was initiated in 2009 when the Biologics Price Competition and Innovation Act (BPCI) was developed by the US Congress [5], allowing the Food and Drug Administration (FDA) to approve the first US biosimilar, Zarxio, in 2015. Before development of the FDA biosimilar path, a series of biological, including follow-on, products had been approved as New Drug Applications by the FDA. Recently (March 2020), some of these products were deemed to be Biologic License Applications (BLAs) and have been removed from FDA’s Orange Book and added to the FDA’s “Purple Book: Lists of Licensed Biological Products with Reference Product Exclusivity and Biosimilarity or Interchangeability Evaluations” [6,7].

**Figure 1:**
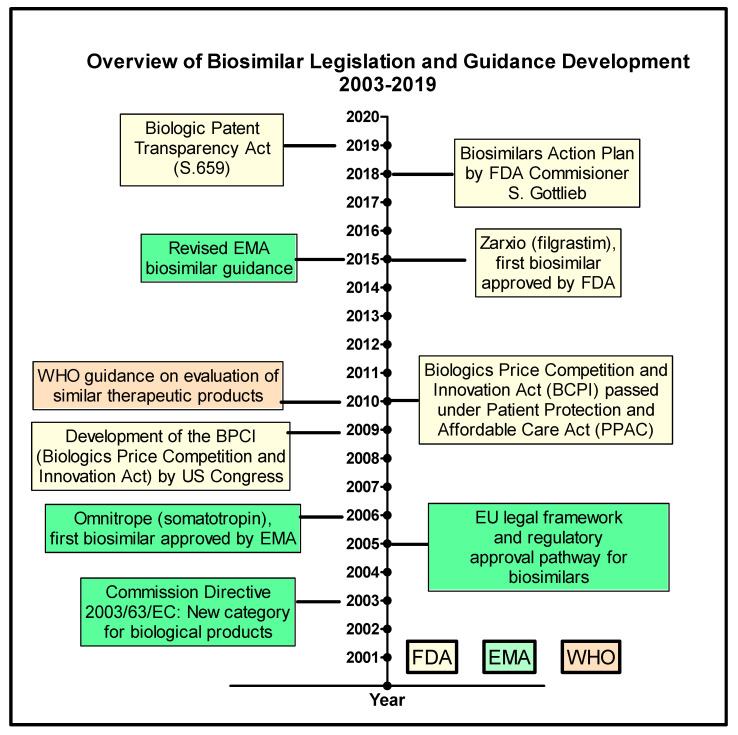
Timeline for the development of World Health Organization (WHO), European Medicines Agency (EMA) and (Food and Drug Administration FDA) legislation and guidance for biosimilars between 2003 and 2019. Data taken from [5,9,10,11].

The regulatory requirements for BS continue to evolve because of the particulars of biosimilar manufacturing as reviewed by Daller [4]. In 2015, a revised EU guideline [8] came into effect and in 2018, the FDA’s Biosimilars Action Plan was developed to support BS development, approval, and commercialization in the US [9]. The chronological evolution of BS legislation and guidelines by the WHO, FDA, and EMA is outlined in Figure 1.

Biosimilar regulations worldwide are based on the concept of demonstrating biosimilarity between the BS molecule and the RP, therefore illustrating a global consensus regarding the approval path for these biotherapeutics. Yet, some differences are found between the approaches taken by the two regulators; the EMA provides product-specific guidelines based on biological classification whereas the FDA, which developed their guidelines much later, takes a case-by-case evaluation approach [4]. Nevertheless, both regulatory agencies have similar requirements for sponsors to demonstrate overall biosimilarity, entailing a step wise approach of ‘head-to-head’ comparisons.

Rigorous comparability exercises between the BS molecule and the RP comprise a “totality of evidence” approach which aims to demonstrate a high degree of similarity between the two molecules, but not to re-generate clinical benefit, safety, or quality data for the BS molecule as this would be a repetition of the RP data. The biosimilar developer focuses mostly on establishing a thorough analytical data package providing an extensive physicochemical and biological profile of the molecule. The last steps of the development process for BSs are the comparative phase I and case-by-case phase III clinical studies that aim to resolve any concerns remaining from the previous steps and establish the similarity of the two molecules in terms of their pharmacokinetic and pharmacodynamic properties. Excellent and detailed overviews of the process and clinical trials have been recently published [12,13]. Recently, the relative value of the comparative pharmacokinetic and efficacy trials in the assessment process has been debated, with an emerging view that demonstrating comparable pharmacokinetics has been, so far, the critical element in successful development and approval of most biosimilars [14,15].

This report aims to provide a perspective of the EU and US biosimilar approvals and their evolution since 2003 when the Commission Directive 2003/63/EC was published (see Figure 1). An additional, secondary objective is to illustrate the typical clinical data (comparative pharmacokinetic and efficacy studies) required to complete the biosimilarity equivalence exercise using the case study of adalimumab biosimilars and the bio-originator, Humira.

## 2. Materials and Methods

### 2.1. Data Mining

Two searches were conducted to generate two different sets of data and produce the results presented and analyzed in this work. The first data mining focused on the current landscape of EMA and FDA biosimilar approvals, whereas the latter gathered information on adalimumab biosimilars, taken as a case study, and particularly on the phase I and III clinical trials conducted for these biosimilars as well as their current label indications and market status within Europe and the US.

#### 2.1.1. Landscape of EMA and FDA Biosimilar Approvals

A medicine search was conducted at the EMA website [16] applying the filter ‘biosimilar’ for the ‘medicine type’ category to find the European Public Assessment Reports (EPARs) for all the centrally authorized biosimilar medicines. This search reflected the status of the EMA repository on the 31 December 2019. The 70 results were reviewed individually to establish the reference medicine, approval date, and marketing authorization holder for each biosimilar. Furthermore, upon analysis of indications, each biosimilar was classified into one of the following therapeutic areas: inflammatory, oncology, immunology, hematology, diabetes mellitus, pituitary pathologies, venous thromboembolism, or osteoporosis. In this study, the term “duplicate Marketing Authorizations (duplicate MAs)” was used for approved biosimilar products corresponding to the same reference molecule and developed by the same sponsor, that had a different brand name and different sets of indications. Please see reference [17] for further detail on this term.

The list of FDA-approved biosimilars was compiled using information from the ‘Biosimilar Product Information’ page on the FDA website [18] with content as current as the 31 December 2019. In addition, a search for each biosimilar on the list was done at ‘Drugs@FDA: FDA-Approved Drugs’ [19] to obtain the corresponding reference product, approval date and company; the approval letter for each biosimilar was used to establish the therapeutic area. This was done by reviewing the indications of the medicine and classifying the product into one of the following therapeutic areas: inflammatory, oncology, immunology, or hematology. Lastly, the market status for each biosimilar was checked in the ‘Biosimilar Approval Status’ table provided by the Biosimilars Review & Report website [20].

#### 2.1.2. Adalimumab Biosimilars Case Study

Adalimumab was chosen as case study as it was the biological reference product with the largest number of biosimilars and therefore, more documentation was available to review. In addition, this case study illustrated both the similarity in the road-to-market through two separate regulatory agencies and the differences between the EU and US markets.

A list including all adalimumab biosimilar molecules was made based upon the data generated through the previous search. The corresponding EMA public assessment reports were scrutinized to gather the details of the clinical studies conducted during phases I and III of their clinical development. Only EPARs were used to this purpose as they provided sufficient, user friendly, well-structured data and because most sponsors behind the adalimumab BS in the US had marketed the same molecules in the EU. Within the assessment reports [16], the Section 2.4.2. “Pharmacokinetics” was studied for each molecule and the data on phase I and III clinical trials were gathered. Lastly, the full list of indications for each adalimumab biosimilar was taken from the EPARs [16] for the EU products, and from the ‘Full Prescribing Information’ document [20] for the US biosimilars.

### 2.2. Data Analysis and Study Limitations.

As patterns in the data set emerged, the data was further classified and analyzed (see Tables in Supplementary Materials). Results and trends were represented in graphs and all data analysis was carried out using Microsoft Excel. The authors acknowledge the following limitations of the study: (a) This work represents a snapshot of the biosimilar approval landscape in December 2019 and no updates subsequent to this date were included in the analysis; (b) To the authors’ knowledge, all relevant information from the FDA and EMA repositories was gathered but the manual data-mining process precludes absolute certainty; (c) This study focused primarily on the patterns of approvals of biosimilars and thus, other crucial factors determining patient access to these medicines were not included [2,21,22,23]; (d) The study focused on biosimilars approved by the EMA centralized procedure and those approved by national competent authorities were not included; (e) Whilst the trends shown are based in the data gathered (available in Supplementary Materials), the authors chose the aspects on which the analysis focused. By providing all the data gathered as supplementary information, other researchers can focus on other aspects and/or reach different conclusions to ours; and finally (f) all results and conclusions have been based on the publicly available information at the EMA and FDA repositories, which represents a fraction, estimated as the most significant, of the dossier submitted to the regulatory bodies.

## 3. Results

### 3.1. EMA and FDA Biosimilar Approvals

The first stage of this work gathered information regarding BS from the EMA and FDA repositories. Tables S1 and S2 in the Supplementary Materials show the full set of raw data, including details for each biosimilar product. At the time of this snapshot, the EMA had received 65 biosimilar marketing authorizations applications (MAAs) including those subsequently granted, withdrawn, or rejected, and the EC (European Commission) had issued a MA (Marketing Authorization) for 55 BS products following a positive opinion by the CHMP (Committee for Medicinal Products for Human Use) of the EMA. The complete list is shown in Table 1 together with the 16 relevant reference products.

Interestingly, some approvals corresponded to the same molecule; these products are duplicate MAs (see reference [17] for definition of duplicate MA) and therefore, were marketed as different medicinal products. One example is Celltrion’s Truxima, Ritemvia, and Blitzima (Table 1), three products with different brand names and different sets of indications, for the same molecule. Normally, this licensing approach reflects a company’s strategy to gain market share. One MAA to the EMA was withdrawn before day 120 of the assessment process by the CHMP, and two MAAs concerning a Roferon A and a human insulin biosimilar were refused. Finally, the EU commission has withdrawn 7 biosimilars from the market following requests by the authorization holders for commercial reasons (see Table S1).

**Figure 2:**
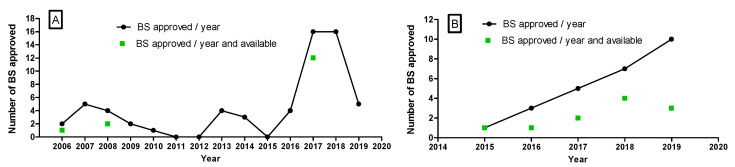
Growth in BS approval in two regulatory areas. (**A**) Yearly EMA biosimilar approvals between 2006 and 2019. (**B**) Yearly FDA biosimilar approvals between 2015 and 2019. The number of products being approved in a given year and available in the market is indicated (green symbols) when it differs from the number approved. Data mined from [16,19].

The growth of the EU biosimilar market between 2006–2019 (see Figure 2) has not been steady and several approval surges were found. For example, the number of approvals clearly increased in 2017 and 2018, with 16 biosimilars approved by the EMA in each year, reflecting the fact that sponsors plan development programs according to expiration dates of patents and exclusivity periods of the bio-originators [22]. In fact, approvals of biosimilars referring to specific RPs are often concentrated in the same year (see Table 1).

The US biosimilar legislation and approval pathway came into force in 2010 and the FDA approved in 2015 the first biosimilar under the BPCI Act (see Figure 1). Table 2 shows the 26 biosimilar medicines approved by the USFDA for nine reference products at the time of this snapshot. However, only 11 of these were currently available on the US market to be prescribed to patients. Table S2 in the Supplementary Materials compiles the raw data with more information on each biosimilar. Two biosimilars have been approved by the EMA corresponding to enoxaparin sodium (Table 1). In contrast, the therapeutic equivalents for Lovenox (enoxaparin sodium) were the object of Abbreviated New Drug Applications (ANDA) suggesting they were considered by the FDA as generic rather than biosimilar products. This classification that has been object of some debate [24].

Despite the challenges experienced by FDA-approved biosimilars in accessing the market, the number of US approvals grew steadily until 2019, as shown in Figure 2. The continuous growth observed reflects the interest of developers in this sector as well as the continuous commitment of the US Government and FDA to supporting a competitive market place through efficient approval of cost-efficient biosimilars. In addition, this rapid growth could also reflect the activity developed to clearing the backlog of product built-up until legislation was developed and patent expirations occurred.

### 3.2. Clinical Evidence in EPARs of Adalimumab Biosimilars

Eight EU-approved biosimilar products, corresponding to five different originators, for the monoclonal antibody adalimumab were found (Table 3), three of which also held an FDA approval. Only one adalimumab biosimilar, Pfizer’s Abrilada, that holds an FDA approval but does not hold an EMA approval, was not included in this case study. The different approaches followed by the five pharmaceutical companies to demonstrate biosimilarity in phase I and III clinical trials were gathered from the assessment reports and analyzed. It was found that the number of clinical trials run and their design were quite similar with only minor differences regarding the patient population selected for the trial and the primary end points used.

The PK profile similarity to the RP is determined in pivotal phase I studies and subsequently, the comparable clinical efficacy is confirmed in phase III supportive studies. Table 4 presents a summary of the clinical pharmacokinetics phase I studies which aimed to demonstrate the similarity between the adalimumab biosimilars and the US- and EU-approved versions of the RP. All studies presented in Table 4 are similarity studies, carried out in a similar number of healthy male and female adults, involving a single 40 mg subcutaneous (SC) injection. Blood samples were taken to measure drug serum concentrations and generate PK profiles, that were later investigated and compared to establish biosimilarity. According to the EMA’s guideline on similar biological medicinal products containing monoclonal antibodies [25], the primary metric in these studies should be the AUC_0–inf_ with C_max_ as a co-primary parameter when adalimumab is administered SC; this advice was followed by all sponsors. As the half-life of adalimumab of is approximately 14 days, the duration of these studies ranged from 63 to 72 days to allow a thorough PK profile generation.

A few findings are worth highlighting in that they illustrate potential challenges in BS development. For example, Sandoz’s first study, GP17-101, failed to demonstrate PK biosimilarity not only between the GP2017 test molecule and the EU-sourced Humira, but also between the EU- and US-sourced RPs. Therefore, the company reconsidered the study design and carried out another trial, GP17-104. The revised design increased the sample size and was restricted to male patients, which were randomized and stratified by body weight. GP17-104 was successful in demonstrating PK profile similarity. Other studies investigated whether the pharmacokinetic profile of the BS could be modified by the administration device used for the SC administration. See, for example, the studies GP17-102 and FKB327–005 conducted for Sandoz’s GP2017 and Mylan’s FKB327 that looked into administration using a pre-filled syringe, a pre-filled auto-injector, or via a vial with disposable syringe (Table 4).

The confirmatory phase III clinical trials are presented in Table 5. The number of phase III clinical trials carried out for biosimilar medicines was reduced compared to reference products. This was expected as BS development is focused on demonstrating similarity to an already-approved medicine rather than establishing a completely new safety and efficacy profile, and because of the extrapolation of indications. Therefore, it is imperative that these similarity studies are carried out in an appropriate population group. The most sensitive population group must be chosen in order to allow an accurate detection of clinically meaningful differences [26]. In the case of adalimumab, the best population were patients with either psoriasis or rheumatoid arthritis. One exception to this approach was found in Amgen’s strategy for their biosimilar molecule ABP 501. This sponsor carried out two different phase III trials (Table 5), one in each of the above-mentioned conditions. All phase III clinical trials found had an equivalence study design, in line with the EMA mAb guidance [25].

These trials aimed to demonstrate that neither the biosimilar molecules nor the reference product, either US- or EU-sourced, were inferior or superior to the other. This was assessed by establishing an equivalence margin, representing a range of clinically acceptable differences in response to treatment. For instance, the study EMR200588-002 measured the PASI75 response rate at week 16 for the Fresenius Kabi molecule MSB11022 compared to the EU-sourced Humira (Table 5). This response involved assessing the percentage of the studied population that achieved at least a 75% improvement in the Psoriasis Area and Severity Index (PASI) [27]. The PASI75 response at week 16 was of similar magnitude for both treatment groups—90% in the MSB11022 group and 92% in the EU-Humira group, giving a 95%CI of (−7.82%,4.16%), which was within the pre-defined equivalence margin of (−18%, 18%) [28].

## 4. Discussion

### 4.1. EMA and FDA Biosimilar Approvals

When analyzing the landscape of biosimilar approvals for the EU and the US, the timeline of regulatory science events should be carefully considered (Figure 1). The EMA was the first regulatory agency to approve a biosimilar in 2006 and to generate guidance documents for biosimilar medicines. Both the WHO and the FDA have built upon the knowledge and vast European experience with biosimilar medicines and have made efforts to harmonize legislation worldwide.

Despite the larger number of European approvals, 55 in 13 years (Table 1), the FDA approval rate is faster compared to the EMA’s initial rate. Between 2006 and 2009, the first four years of biosimilar approvals, the EMA approved 13 biosimilars, whilst the FDA approved 16 biosimilars between 2015 and 2018, the US first four years of BS approvals. The implementation of an approval pathway which built upon the legislative and expertise foundation laid by the EMA could explain the faster approval rate for the FDA. In contrast, the EMA’s approach could have been more cautious, as it was the first time that legislation and guidelines were developed to ensure ‘safe and efficacious’ [4] biosimilar approvals.

**Figure 3:**
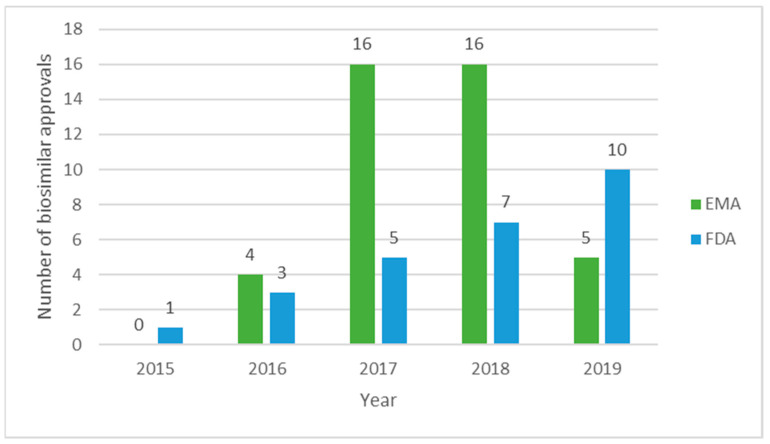
EMA and FDA biosimilar approvals between 2015 and 2019. Data taken from [12,18].

Figure 3 shows the approval trend for both agencies during the period 2015–2019. The EMA approved a record of 16 biosimilars in both 2017 and 2018, with fewer approvals in 2019, while the FDA is growing their biosimilar portfolio at a current, steady pace, yet the overall number of approvals is fewer compared to the EMA’s.

**Figure 4:**
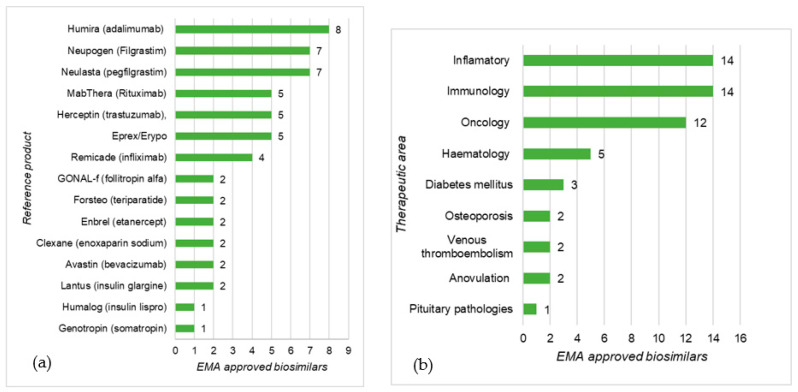
EMA-approved biosimilars distribution by reference product (**a**) and therapeutic area (**b**); data taken from [16].

**Figure 5:**
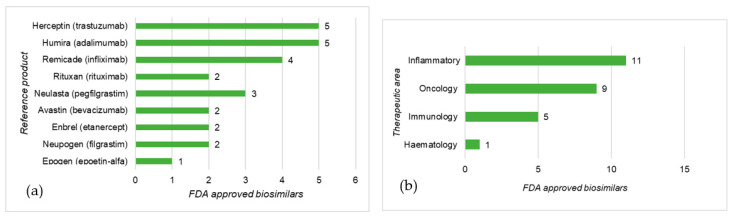
FDA-approved biosimilars distribution by reference product (**a**) and therapeutic area (**b**); data taken from [18,19].

There is a close link between therapeutic areas, prevalence, and the cost of diseases and biosimilar development. According to Pelechas [29] rheumatic diseases affect 52.5 million Americans and cost the American healthcare system $128 billion annually. In EU countries, the cost for treating and supporting patients with RA is estimated to be €25.1 billion [29], this scenario favors the development of BS such as SB-4 Etanercept for this condition. Because most of this cost results from expensive treatments with biologicals, biosimilars represent a cost-saving option that is very attractive to healthcare systems. Rheumatic diseases are inflammatory conditions and, as shown by Figure 4 and Figure 5, most EMA- and FDA-approved biosimilars are used for inflammatory conditions. Immunology and oncology are also therapeutic areas that represent a financial burden on the healthcare systems and in which biotherapies have a great impact on patient outcomes and therefore, there is a pressing need for biosimilar alternatives.

In general, similar trends in the distribution of biosimilars per therapeutic area were observed for the EU and US areas, although a more extensive biosimilar portfolio, covering more therapeutic areas, was found for the EU. In both cases, the RPs used in inflammatory diseases, immunology, and oncology were those with the most biosimilar molecules available as they represented the most profitable areas for sponsors of biosimilar development.

### 4.2. Clinical Evidence Package Published in EPARs for Adalimumab Biosimilars

The clinical development process of a biosimilar medicine is designed to meet the licensing criteria set out by regulatory agencies. This explains the similarity in the approaches taken to demonstrate biosimilarity by the five different pharmaceutical companies that developed adalimumab-biosimilar molecules. Indeed, the EMA’s ‘Guideline on similar biological medicinal products containing monoclonal antibodies—non-clinical and clinical issues’ [25] was used to guide the sponsors’ clinical development programs. This guideline provides scientific advice on designing a study that it is sensitive enough to detect any clinically meaningful differences between the biosimilar and the bio-originator. To meet this end, aspects such as patient population, primary endpoints, and study design (equivalence vs. non-inferiority) must be carefully considered by the sponsors.

Results from these studies are used to generate a data package that establishes the quality, safety and efficacy profile for the biosimilar molecule after being studied in one indication that can be extrapolated to other indications of the reference medicine upon adequate justification, on a case-by-case basis. The latter represents a highly challenging area that requires further research, as many healthcare professionals are trying to grasp the basis of extrapolation in order to determine the suitability of biosimilars for their patients [23]. According to Barbier [30], the regulatory concept of extrapolation becomes a hurdle for healthcare professionals who need to decide on the use of a biosimilar for different pathological cancer conditions to that used for regulatory approval and for the same indication but different line (disease-stage) patients.

Intuitively, it would seem that most manufacturers would seek extrapolation to all indications of the reference medicine for their approved BS. However, there are some cases (see Table 6), in which BS with reduced labels were licensed by the EMA under a duplicate marketing authorization [17] because of patent protection. The commercialization of products with restricted licenses on the EU market is a regulatory-affairs strategy that aims to work around patents and exclusivity periods of bio-originators in certain countries. For example, Abbvie’s Humira, the bio-originator of adalimumab, has 12 labelled indications, as shown in Table 6.

Sandoz has developed an adalimumab molecule, GP2017, that is currently commercialized in Europe under three different brand names—Hyrimoz, which has the full label, and Hefiya, and Halimatoz which have a restricted license (Table 6). Although these restrictions allow market penetration that otherwise would not possible, this plethora of names may lead to confusion among prescribers regarding product choice and avoidance of off-licensing prescribing [23]. For the time being, this is a problem specific to the EU market which in December 2020, had eight marketed forms of the five EMA-approved adalimumab biosimilars as shown in Table 6 (note that Kromeya was withdrawn in February 2020).

In contrast, the FDA-approved adalimumab biosimilars have not yet been able to access the US market. Although, the FDA has approved three different biosimilar molecules (Table 3), the patents protecting the bio-originator Humira prevent the market entry of adalimumab biosimilars. In 2018, Humira was the world’s best-selling drug, achieving a yearly revenue of $20.5 billion global sales [31]. It is not surprising that a company tries to maintain the highest share in the US market by designing a complex net of exclusivity periods and patents to protect Humira. The absence of a biosimilar product offering prescribers an alternative, more-economic choice, makes Humira very profitable. The first adalimumab biosimilars will not be marketed in the US until January 2023, when Amgen will launch Amjevita after reaching a global resolution ending patent litigations with Abbvie [32]. Sandoz reached a settlement with Abbvie involving royalty payments [33] that grants Sandoz a non-exclusive license to AbbVie’s intellectual property relating to Humira, and in the US, the license period for Hyrimoz will begin on September 30, 2023. By 2023, Humira will have enjoyed 20 years of US market exclusivity. This aspect causes striking differences between the EU and US biosimilar markets: the EU will have seen at least eight Adalimumab biosimilars commercialized before the first one is marketed in the US. Briefly, these market and exclusivity issues [22] can represent the critical hurdle for the health-care sector to benefit from some biosimilar therapeutics and so far, it would seem that EU patients are likely to see the benefits of BS treatments much quicker than their US counterparts.

Despite this unfortunate situation from the patients’ and healthcare providers’ perspective, the US government and the FDA are committed to supporting not only biosimilar approval but also market access and therefore, the Biosimilar Action Plan (BAP) was published in 2018 [9]. The BAP focuses in four areas: (a) improving the efficiency of the biosimilar and interchangeable product development and approval process, (b) maximizing scientific and regulatory clarity for the biosimilar product development community, (c) developing effective communications to improved understanding of biosimilar by patients, clinicians, and payers, and (d) supporting market competition by reducing gaming of FDA requirements or other attempts to unfairly delay competition [9]. In this context, “gaming” is understood as developing strategies in order to work around FDA requirements to extend exclusivity periods and profit from market exclusivity for longer. As illustrated in the report ‘Biosimilars in the USA’ [1], the introduction of biosimilars in the US market is more difficult due to the complexity of their healthcare system and financing.

Because of the continuous evolution of the biosimilar landscape and its expected growth in the next five years, it will be important to continue research in this field to monitor patients’ access to these innovative treatments and the benefits they bring to patient outcomes and healthcare systems.

## 5. Conclusions

Despite an increasing number of biosimilars being developed by numerous pharmaceutical companies and authorized by regulatory bodies worldwide, the full economic and clinical benefits of these medicines will only be determined once they become more widely available in the market and used in clinical practice. In the past 13 years, the EMA has approved 55 biosimilars and during the past 5 years, the FDA has approved 26 biosimilars, with fewer than 50% currently being commercialized on the US market.

Market penetration and uptake by prescribers is essential for biosimilars to impact patient health and healthcare systems. While hurdles to market penetration in the US are currently being tackled by the Biosimilar Action Plan, understanding the different clinical development pathway these medicines undergo is of utmost importance to prescribers. The five adalimumab biosimilars studied by this report went through comparative phase I studies and phase III equivalence studies in RA and psoriasis in order to meet the EU and US regulatory requirements. An important difference was found between the EU and the US markets regarding commercialization of adalimumab biosimilars. The EU market has seen eight biosimilar products of adalimumab, while the US will be able to commercialize their first one only in 2023 due to the bio-originator’s US market exclusivity.

Overall, the considerable interest in biosimilar development from the pharmaceutical industry alongside the development of biosimilar regulations worldwide has led to a growing biosimilar market. In spite of the progress that has been made so far, further research is needed to assess the future real impact of these medicines on public health and patient outcomes, as BS market penetration continues to develop globally.

## Figures and Tables

**Table 1:** European biosimilar approvals: The available biosimilar (BS) products at the time of this report with corresponding approval dates are listed for each reference product. Note that withdrawn products are not listed in this Table but are available in the Supplementary Materials. Note that Kromeya was withdrawn from the market in February 2020. Data taken from [16].

Reference Product	Biosimilar Name	Approval Date	Biosimilar Name		Approval Date
MabThera (rituximab)	Truxima (rituximab)	17 February 2017	Rixathon (rituximab)		15 June 2017
	Ritemvia (rituximab)	13 July 2017	Riximyo (rituximab)		15 June 2017
	Blitzima (rituximab)	13 July 2017			
Eprex/Erypo (epoetin alfa)	Epoetin Alfa Hexal	27 August 2007			
	Binocrit (epoetin alfa)	28 August 2007			
	Abseamed (epoetin alfa)	27 August 2007			
Eprex/Erypo (epoetin zeta)	Retacrit (epoetin zeta)	18 December 2007			
	Silapo (epoetin zeta)	18 December 2007			
Herceptin (trastuzumab)	Ontruzant (trastuzumab)	15 November 2017	Kanjinti (trastuzumab)		16 May 2018
	Trazimera (trastuzumab)	26 July 2018	Ogivri (trastuzumab)		12 December 2018
	Herzuma (trastuzumab)	8 February 2018			
Neupogen (filgrastim)	Nivestim (filgrastim)	7 June 2010	Grastofil (filgrastim)		17 October 2013
	Filgrastim Hexal (filgrastim)	6 February 2009	Tevagrastim (filgrastim)		15 September 2008
	Zarzio (filgrastim)	6 February 2009	Ratiograstim (filgrastim)		15 September 2008
	Accofil (filgrastim)	17 September 2014			
Neulasta (pegfilgrastim)	Pelmeg (pegfilgrastim)	20 November 2018	Ziextenzo (pegfilgrastim)		22 November 2018
	Udenyca (pegfilgrastim)	21 September 2018	Grasustek (pegfilgrastim)	20 June 2019	
	Fulphila (pegfilgrastim)	20 November 2018	Cegfila (pegfilgrastim)	19 December 2019	
	Pelgraz (pegfilgrastim)	21 September 2018			
Remicade (infliximab)	Remsima (infliximab)	10 September 2013	Zessly (infliximab)		18 May 2018
	Inflectra (infliximab)	9 September 2013			
	Flixabi (infliximab)	26 May 2016			
Enbrel (etanercept)	Benepali (etanercept)	13 January 2016			
	Erelzi (etanercept)	23 June 2017			
GONAL-f (follitropin alfa)	Ovaleap (follitropin alfa)	27 September 2013			
	Bemfola (follitropin alfa)	26 March 2014			
Avastin (bevacizumab)	Zirabev (bevacizumab)	14 February 2019			
	Mvasi (bevacizumab)	15 January 2018			
Humira (adalimumab)	Hefiya (adalimumab)	26 July 2018	Amgevita (adalimumab)		21 March 2017
	Kromeya (adalimumab)	2 April 2019	Idacio (adalimumab)		2 April 2019
	Imraldi (adalimumab)	24 August 2017	Halimatoz (adalimumab)		26 July 2018
	Hyrimoz (adalimumab)	26 July 2018	Hulio (adalimumab)		16 September 2018
Lantus (insulin glargine)	Semglee (insulin glargine)	23 March 2018			
	Abasaglar (insulin glargine)	09 September 2014			
Humalog (insulin lispro)	Insulin lispro Sanofi (insulin lispro)	18 July 2017			
Genotropin (somatropin)	Omnitrope (somatropin)	12 April 2006			
Clexane (enoxaparin sodium)	Thorinane (enoxaparin sodium)	14 September 2016			
	Inhixa (enoxaparin sodium)	15 September 2016			
Forsteo (teriparatide)	Terrosa (teriparatide)	4 January 2017			
	Movymia (teriparatide)	11 January 2017			

**Table 2:** FDA biosimilar approvals: All approved BS products at the time of this report, with corresponding approval date and market status are listed for each reference product. Data taken from [18,19].

Reference Product	Biosimilar Name	Approval Date	Market Status
Avastin (bevacizumab)	Mvasi (bevacizumab-awwb)	14 September 2017	Available
	Zirabev (bevacizumab-bvzr)	27 June 2019	Available
Enbrel (etanercept)	Erelzi (etanercept-szzs)	30 August 2016	Not available
	Eticovo (etanercept-ykro)	25 April 2019	Not available
Epogen (epoetin-alfa)	Retacrit (epoetin alfa-epbx)	15 May 2018	Available
Herceptin (trastuzumab)	Ogivri (trastuzumab-dkst)	1 December 2017	Not available
	Herzuma (trastuzumab-pkrb)	14 December 2018	Not available
	Ontruzant (trastuzumab-dttb)	18 January 2019	Not available
	Trazimera (trastuzumab-qyyp)	11 March 2019	Not available
	Kanjinti (trastuzumab-anns)	13 June 2019	Available
Humira (adalimumab)	Amjevita (adalimumab-atto)	23 September 2016	Not available
	Cyltezo (adalimumab-adbm)	25 August 2017	Not available
	Hyrimoz (adalimumab-adaz)	30 October 2018	Not available
	Hadlima (adalimumab-bwwd)	23 July 2019	Not available
	Abrilada (adalimumab-afzb)	15 November 2019	Not available
Neulasta (pegfilgrastim)	Fulphila (pegfilgrastim-jmdb)	4 June 2018	Available
	Udenyca (pegfilgrastim-cbqv)	2 November 2018	Available
	Ziextenzo (pegfilgastrim-bmez)	4 November 2019	Available
Neupogen (filgrastim)	Zarxio (filgrastim-sndz)	6 March 2015	Available
	Nivestym (filgrastim-aafi)	20 July 2018	Available
Remicade (infliximab)	Inflectra (infliximab-dyyb)	5 April 2016	Available
	Ixifi (infliximab-qbtx)	13 December 2017	Not available
	Renflexis (infliximab-abda)	21 April 2017	Available
	Avsola (infliximab-axxq)	6 December 2019	Not available
Rituxan (rituximab)	Truxima (rituximab-abbs)	28 November 2018	Not available
	Ruxience (rituximab-pvvr)	23 July 2019	Not available

**Table 3:** The number (N) of phase I and phase III studies alongside the condition investigated in Phase III trials for each product as stated by European Public Assessment Reports (EPARs). Data gathered from [16,18].

Company	Molecule	Brand Name	Approval Date		Phase I Studies (N)	Phase III Studies	
			EU	FDA		(N)	Investigated Conditions
Sandoz	GP2017	EU Hyrimoz (adalimumab)	July 2018	NA	3	1	Plaque psoriasis
		US Hyrimoz (adalimumab-adaz)	NA	October 2018			
		Halimatoz (adalimumab)	July 2018	NA			
		Hefiya (adalimumab)	July 2018	NA			
Amgen	ABP 501	Amgevita (adalimumab)	March 2017	NA	1	2	RA and psoriasis
		Amjevita (adalimumab-atto)	NA	September 2016			
Samsung Bioepis NL B.V.	SB5	Imraldi (adalimumab)	August 2017	NA	1	1	RA
		Hadlima (adalimumab-bwwd)	NA	July 2019			
Fresenius Kabi Deutschland GmbH	MSB11022	Kromeya (adalimumab)	April 2019	NA	1	1	Plaque psoriasis
		Idacio (adalimumab)	April 2019	NA			
Mylan S.A.S.	FKB327	Hulio (adalimumab)	September 2018	NA	2	1	RA

**Table 4:** Phase I clinical trials for the adalimumab biosimilar molecules. PFS, pre-filled syringe; AI, pre-filled auto injector; Data taken from [16]. * N = number of participants enrolled, initial number of patients enrolled in the study, not accounting for patient discontinuation.

Company	Molecule	Study Number	Duration (Days)	N *	Comparing	Comparer	Primary PK Endpoints
Amgen	ABP 501	20110217	63	203	PK profile	Adalimumab EU and US	AUC_0–inf_, AUC_0–t_, C_max_
Sandoz	GP2017	GP17-104	72	318	PK profile	Adalimumab EU and US	AUC_0–inf_, C_max_
		GP17-101	72	219	PK profile	Adalimumab EU and US	AUC_0–inf_, AUC_0–last_, C_max_
		GP17-102	72	108	PK profile	AI vs. PFS administration	AUC_0–360 h,_ C_max_
Samsung Bioepis NL B.V.	SB5	SB5-G11-NHV	70	189	PK profile	Adalimumab EU and US	EMA: AUC_inf_, C_max_ FDA: AUC_inf_, AUC_last_, C_max_
Fresenius Kabi Deutschland GmbH	MSB11022	EMR200588-001	70	233	PK profile	Adalimumab EU and US	AUC_0–inf_, AUC_0–t_, C_max_
Mylan S.A.S.	FKB327	FKB327-001	64	180	PK profile	Adalimumab EU and US	AUC_0–last_, C_max_
		FKB327-005	64	129	Relative bioavailability	Delivery via vial, PFS and AI	AUC_0–t_, AUC_0–inf_, C_max_

**Table 5:** Phase III clinical trials conducted for adalimumab biosimilars; data taken from [16]. * N = number of participants enrolled, initial number of patients enrolled in the study, not accounting for patient discontinuation.

Molecule	Study Number	Studied Population	N *	Comparison	Primary Efficcay Endpoint	Equivalence Margin	Primary Endpoint Results	95% CI
ABP 501	20120262	Adults with moderate to severe RA	526	ABP 501 vs. adalimumab (US)	Risk ratio of ACR20 at week 24	[0.738; 1/0.738]	ACR20 response rate 74.6% vs. 72.4%, risk ratio of ACR20 ABP501 vs. Humira 1.039	(0.954, 1.133)
	20120263	Adults with moderate to severe psoriasis	350	ABP 501 with adalimumab (EU)	PASI% improvement from baseline at week 16	[−15%; 15%]	PASI% improvement 80.91%(ABP 501) vs. 83.06% (Humira)–difference in response −2.18	(−7.39, 3.02)
GP2017	GP17-301	Male and female patients with moderate to severe chronic plaque-type psoriasis	465	GP2017 vs. EU-Humira and US-Humira	PASI75 response rate at week 16	[−18%; 18%]	66.8% for GP2017 and 65.0% for Humira	(−7.46, 11.15)
SB5	SB5-G31-RA	Adults with moderate to severe rheumatoid arthritis despite methrotexate	544	SB5 vs. EU Humira	ACR20 response rate at week 24	[−15%; 15%]	ACR20 response rate at week 24 68.0% (183/269) SB5 and 67.4% (184/273) Humira	(−7.83, 8.13)
MSB11022	EMR200588-022	Patients with moderate to severe chronic plaque psoriasis	382	MSB11022 vs. EU-approved Humira	PASI75 at week 16	[−18%; 18%]	PASI75 response at week 16: 90% in MSB1102 group and 92% in EU-Humira group	(−7.82, 4.16)
FKB327	FKB327-022 followed by FKB327-003	Rheumatoid arthritis patients inadequately controlled on methotrexate	680	FKB327 vs. US-Humira	ACR20 response rate at week 24	[−13%; 13%]	270 patients (74.4%) in the FKB327 treatment group achieved an ACR20 response at week 24 compared to 271 patients (75.7%)	(−7.6, 5.0)

**Table 6:** Indication, label type (full/reduced), and regulatory jurisdiction for the reference product Humira and approved adalimumab-biosimilar products. Data taken from [16,19]. Note that Kromeya was withdrawn from the market in February 2020.

Pharmaceutical Company	ABBVIE		SANDOZ				AMGEN		Samsung Bioepis NL B.V.		Fresenius Kabi Deutchsland		MYLAN
Indication	EU Humira	US Humira	EU Hyrimoz	US Hyrimoz	Hefiya	Halimatoz	Amgevita	Amjevita	Imraldi	Hadlima	Kromeya	Idacio	Hulio
Rheumatoid Arthritis	yes	yes	yes	yes	no	yes	yes	yes	yes	yes	yes	yes	yes
Juvenile idiopathic arthitis	yes	yes	yes	yes	yes	yes	yes	yes	yes	yes	yes	yes	yes
Ankylosing spondylitis (AS)	yes	yes	yes	yes	yes	yes	yes	no	yes	yes	yes	yes	yes
Psoriatic Arthritis	yes	yes	yes	yes	yes	yes	yes	yes	yes	yes	yes	yes	yes
Plaque Psoriasis	yes	yes	yes	yes	yes	yes	yes	yes	yes	yes	yes	yes	yes
Paediatric Plaque Psoriasis	yes	no	yes	no	yes	yes	yes	no	yes	no	yes	yes	yes
Hidradenitis suppurativa (HS)	yes	yes	yes	no	yes	yes	yes	no	yes	no	no	yes	yes
Crohn’s Disease	yes	yes	yes	yes	no	no	yes	yes	yes	yes	yes	yes	yes
Pediatric Crohn’s Disease	yes	yes	yes	no	no	no	yes	no	yes	no	yes	yes	yes
Ulcerative Colitis	yes	yes	yes	yes	no	no	yes	yes	yes	no	yes	yes	yes
Uveitis	yes	yes	yes	no	yes	yes	yes	no	yes	no	yes	yes	yes
Paediatric uveitis	yes	yes	yes	no	yes	yes	yes	no	yes	no	yes	yes	yes
LABEL	Full	Reduced	Full	Reduced	Reduced	Reduced	Full	Reduced	Full	Reduced	Reduced	Full	Full
Number of indications	12	11	12	7	8	9	12	6	12	6	11	12	12
Regulatory jurisdiction	EU	US	EU	US	EU	EU	EU	US	EU	US	EU	EU	EU

## Data Availability

The data presented in this study are available in the Supplementary Material provided.

## Supplementary Materials

The following are available online at https://www.mdpi.com/1999-4923/13/1/48/s1, Excel file comprising Excel data sheets providing the following raw data. Table S1: European Medicines Agency Biosimilar Marketing Authorizations Applications, Table S2: Food and Drug Administration Biosimilar Marketing Authorization Applications and Market Status.

## References

[1] Mehr S., Brook R. (2019). Biosimilars in the USA: Will new efforts to spur approvals and access spur uptake and cost savings?. Pharm. Med..

[2] Troein P., Newton M., Pate J., Scott K. (2019). The Impact of Biosimilar Competition in Europe. European Commission. IQVIA Report. https://ec.europa.eu/docsroom/.

[3] WHO Questions and Answers: Similar Biotherapeutic Products. https://www.who.int/biologicals/QA_for_SBPs_HK_12_Dec_2017_(2).pdf22.

[4] Daller J. (2016). Biosimilars: A consideration of the regulations in the United States and European Union. Regul. Toxicol. Pharm..

[5] Wang J., Chow S. (2012). On the regulatory approval pathway of biosimilar products. Pharmaceuticals.

[6] U.S. Food and Drug Administration List of Approved NDAs for Biological Products That Were Deemed to Be BLAs on 23 March 2020. https://www.fda.gov/media/119229/download.

[7] U.S. Food and Drug Administration Purple Book Database of Licensed Biological Products. https://purplebooksearch.fda.gov/.

[8] Guideline on Similar Biological Medicinal Products. CHMP/437/04 Rev 1. EMA. https://www.ema.europa.eu/en/documents/scientific-guideline/guideline-similar-biological-medicinal-products-rev1_en.pdf.

[9] Biosimilars Action Plan: Balancing Innovation and Competition. https://www.fda.gov/media/114574/download.

[10] Multidisciplinary: Biosimilar—EMA. https://www.ema.europa.eu/en/human-regulatory/research-development/scientific-guidelines/multidisciplinary/multidisciplinary-biosimilar.

[11] World Health Organization Similar Biotherapeutic Products. https://www.who.int/biologicals/biotherapeutics/similar_biotherapeutic_products/en/.

[12] Bellinvia S., Fraser Cummings J.R., Ardern-Jones M.R., Edwards C.J. (2019). Adalimumab biosimilars in Europe: An overview of the clinical evidence. BioDrugs.

[13] Moore T.J., Mouslim M.C., Blunt J.L., Alexander G.C., Shermock K.M. (2020). Assessment of availability, clinical testing, and US Food and Drug Administration review of biosimilar biologic products. JAMA Intern. Med..

[14] Schiestl M., Ranganna G., Watson K., Jung B., Roth K., Capsius B., Trieb M., Bias P., Maréchal-Jamil J. (2020). The path towards a tailored clinical biosimilar development. BioDrugs.

[15] Wolff-Holz E., Tiitso K., Vleminck C., Weise M. (2019). Evolution of the EU biosimilar framework: Past and future. BioDrugs.

[16] Medicines European Medicines Agency. https://www.ema.europa.eu/en/medicines/field_ema_web_categories%253Aname_field/Human/ema_group_types/ema_medicine/field_ema_med_status/authorised-36/ema_medicine_types/field_ema_med_biosimilar/search_api_aggregation_ema_medicine_types/field_ema_med_biosimilar.

[17] European Commission Handling of Duplicate Marketing Authorization Applications. Health and Consumers Directorate-General. https://ec.europa.eu/health/sites/health/files/files/latest_news/2011_09_duplicates_note_upd_01.pdf.

[18] U.S. Food and Drug Administration Biosimilar Drug Information. https://www.fda.gov/drugs/biosimilars/biosimilar-product-information.

[19] Drugs@FDA: FDA-Approved Drugs. https://www.accessdata.fda.gov/scripts/cder/daf/.

[20] Biosimilars Approved in the US and Filed for FDA Approval. https://biosimilarsrr.com/us-biosimilar-filings/.

[21] Rathore A.S., Vulto A.G., Stevenson J.G., Shah V.S. (2019). Challenges with successful commercialization of biosimilars. BioPharm Int..

[22] Rothwell Figg’s Biologics and Biosimilars Group, Rothwell Figg’s Biosimilars Law Bulletin. How the U.S. Compares to Europe on Biosimilar Approvals and Products in the Pipeline. https://www.biosimilarsip.com/2020/10/12/how-the-u-s-compares-to-europe-on-biosimilar-approvals-and-products-in-the-pipeline-5/.

[23] Sarzi-Puttinia P., Marotto D., Caporalic R., Galeazzid M., Atzenie F., Hamarf A., Soósf B., Szekanecz Z. (2019). Biosimilars vs. originators: Are they the same?. Autoimmun. Rev..

[24] Enoxaparin Biosimilar or Not. Generics and Biosimilars Initiative. http://www.gabionline.net/Biosimilars/General/Enoxaparin-biosimilar-or-not#:~:text=A%20biosimilar%20of%20the%20low%20molecular%20weight%20heparin%2C,whether%20this%20was%20really%20a%20biosimilar%20or%20not.

[25] Guideline on Similar Biological Medicinal Products Containing Monoclonal Antibodies—Non-Clinical and Clinical Issues. https://www.ema.europa.eu/en/documents/scientific-guideline/guideline-similar-biological-medicinal-products-containing-monoclonal-antibodies-non-clinical_en.pdf.

[26] Age Kos I., Azevedo V., Neto D., Kowalski S. (2018). The biosimilars journey: Current status and ongoing challenges. Drugs Context.

[27] Smith C.H., Yiu Z.Z.N., Bale T., Burden A.D., Coates L.C., Edwards W., MacMahon E., Mahil S., McGuire A., Murphy R. (2020). British Association of Dermatologists guidelines for biologic therapy for psoriasis 2020: A rapid update. Br. J. Dermatol..

[28] Kromeya CHMP Assessment Report. https://www.ema.europa.eu/en/documents/assessment-report/kromeya-epar-public-assessment-report_en.pdf.

[29] Pelechas E., Drosos A. (2019). Etanercept biosimilar SB-4. Expert Opin. Biol. Ther..

[30] Barbier L., Declerck P., Simoens S., Neven P., Vulto A., Huys I. (2019). The arrival of biosimilar monoclonal antibodies in oncology: Clinical studies for trastuzumab biosimilars. Br. J. Cancer.

[31] Speights K. Biggest Blockbuster Drugs of the Future. The Motley Fool. https://www.fool.com/investing/2019/06/15/5-biggest-blockbuster-drugs-of-the-future.aspx.

[32] Amgen’s Adalimumab Biosimilar Will Only Be Launched in US in 2023 Generics and Biosimilars Initiative. http://gabionline.net/Biosimilars/News/Amgen-s-adalimumab-biosimilar-will-only-be-launched-in-US-in-2023#:~:text=The%20settlement%20means%20that%20Amgen%E2%80%99s%20adalimumab%20biosimilar%20will,launched%20in%20the%20US%20on%2031%20January%202023.

[33] Sandoz Announces Global Resolution of Biosimilar Adalimumab Patent Disputes, Securing Patient Access. https://www.sandoz.com/news/media-releases/sandoz-announces-global-resolution-biosimilar-adalimumab-patent-disputes.

